# Editorial: Phasor analysis for fluorescence lifetime data

**DOI:** 10.3389/fbinf.2024.1375480

**Published:** 2024-02-06

**Authors:** Ruofan Cao, Yide Zhang, Jessica Houston

**Affiliations:** ^1^ Florida Research and Innovation Center, Cleveland Clinic, Port St. Lucie, FL, United States; ^2^ Caltech Optical Imaging Laboratory, Andrew and Peggy Cherng Department of Medical Engineering, Department of Electrical Engineering, California Institute of Technology, Pasadena, CA, United States; ^3^ Chemical and Materials Engineering, New Mexico State University, Las Cruces, NM, United States

**Keywords:** Phasor, Fluorescence, Lifetime, imaging, Biosensing

Fluorescence lifetime measurements are valuable for many scientific studies that span biological (Berezin and Achilefu, 2010), biomedical (Marcu, 2012), biochemical (Datta et al., 2020), and biophysical (Geacintov, 1987) domains. The quantitative nature of fluorescence lifetime data, which are largely independent of fluorophore concentration, provides invaluable metrics for understanding intracellular changes such as molecular interactions (Sun et al., 2011), metabolic fluctuations, and intracellular microenvironmental properties. Moreover, many advanced biphotonic techniques, including polarization imaging (Levitt et al., 2009), super-resolution imaging (Wang et al., 2018; Zhang et al., 2018), and flow cytometry (Cao et al., 2016) have been augmented so as to measure fluorescence lifetimes. However, the interpretation of lifetime data can be confounding, because of the intensive data processing, regression, and fitting that is necessary, as well as the need for component analyses. To address this challenge, a simple and graphical vector-based method, known widely as “phasor plotting,” has become mainstream. Owing to the popularity and effectiveness of phasor analyses, this Research Topic was conceived, and a collection of articles was compiled that describe unique ways in which fluorescence lifetime analyses are conducted by vectorizing frequency-domain and time-domain lifetime data.

We feature five original research articles that cover recent advances in phasor plotting and its applications for biosensing and imaging. Expectedly, machine learning (ML) takes center stage, because of the ease that ML brings when enhancing image processing performance. The contribution by Hu et al. introduces an ML approach to predict cellular phenotypes from fluorescence lifetime imaging microscopy (FLIM) data, comparing phasor analysis with biexponential decay curve fitting using Random Forest Tree algorithms. The study validates the accuracy of phasor analysis, emphasizing its benefits, such as handling an unknown number of lifetime components and computational efficiency.

In other contributions, ML extends beyond pattern recognition to improve image quality and extract valuable information from original data. Mannam et al. propose a novel approach using pre-trained Convolutional Neural Networks (CNNs) to denoise FLIM images and enhance phasor accuracy for segmenting different fluorophores. By utilizing pre-trained CNN models like DnCNN and Noise2Noise, trained on extensive fluorescence image datasets, the method eliminates the need to train a model from scratch for FLIM data. The denoised FLIM data is then subjected to K-means clustering on phasor plots to identify different fluorophores, demonstrating improved noise removal, signal-to-noise ratio (SNR), and segmentation accuracy compared to raw noisy data.

Addressing the need for user-friendly FLIM processing, Tan et al. present an open-source program for real-time FLIM analysis. Integrated as a Napari plugin, this program seamlessly interfaces with other open-source FLIM data acquisition and analysis tools, such as OpenScan, Micro-Manager, and FLIMLib. The modular design enables real-time FLIM acquisition, display, and analysis, overcoming post-acquisition workflow limitations in Time-Correlated Single Photon Counting (TCSPC)-based FLIM systems.

Beyond technological discussions, the research collection explores phasor plot applications in biomedical research. Xue et al. employ FLIM and phasor plots to noninvasively monitor and characterize metabolic and structural changes at the cellular level during retinal organoid (RtOg) development and differentiation. The study offers valuable insights for live RtOg characterization, suggesting a potent tool for screening and quality control in RtOg tracing.

The application of phasor plots extends to clinical settings, as demonstrated by Poulon et al. In their study, phasor plots are used to discriminate between brain tumors and healthy tissue (cortex) and distinguish primary (glioblastoma) from secondary (metastasis) tumors. The method’s potential for real-time analysis in the operating room might aid doctors in identifying tumor margins swiftly and directly.

This Research Topic is important for the broad field of biological sensing with fluorescence lifetime as a parameter. It is evident that phasor plotting approaches have experienced rapid development in recent years. Their unique capabilities as a tool for easing FLIM processing in both research and clinical settings open new avenues for scientists and doctors alike. The contributions of the research authors and the constructive input from reviewers provide salient examples of the importance of phasor methods. , Aspiringly, this Research Topic serves as a valuable reference, so as to encourage cross-disciplinary collaborations and catalyze future advancements in imaging technologies across biological and health sciences.
